# Detection of segregation distortion loci in triticale (x *Triticosecale *Wittmack) based on a high-density DArT marker consensus genetic linkage map

**DOI:** 10.1186/1471-2164-12-380

**Published:** 2011-07-28

**Authors:** Katharina V Alheit, Jochen C Reif, Hans Peter Maurer, Volker Hahn, Elmar A Weissmann, Thomas Miedaner, Tobias Würschum

**Affiliations:** 1 State Plant Breeding Institute, University of Hohenheim, 70593 Stuttgart, Germany; 2 Saatzucht Dr. Hege GbR Domäne Hohebuch, 74638 Waldenburg, Germany

## Abstract

**Background:**

Triticale is adapted to a wide range of abiotic stress conditions, is an important high-quality feed stock and produces similar grain yield but more biomass compared to other crops. Modern genomic approaches aimed at enhancing breeding progress in cereals require high-quality genetic linkage maps. Consensus maps are genetic maps that are created by a joint analysis of the data from several segregating populations and different approaches are available for their construction. The phenomenon that alleles at a locus deviate from the Mendelian expectation has been defined as segregation distortion. The study of segregation distortion is of particular interest in doubled haploid (DH) populations due to the selection pressure exerted on the plants during the process of their establishment.

**Results:**

The final consensus map, constructed out of six segregating populations derived from nine parental lines, incorporated 2555 DArT markers mapped to 2602 loci (1929 unique). The map spanned 2309.9 cM with an average number of 123.9 loci per chromosome and an average marker density of one unique locus every 1.2 cM. The R genome showed the highest marker coverage followed by the B genome and the A genome. In general, locus order was well maintained between the consensus linkage map and the component maps. However, we observed several groups of loci for which the colinearity was slightly uneven. Among the 2602 loci mapped on the consensus map, 886 showed distorted segregation in at least one of the individual mapping populations. In several DH populations derived by androgenesis, we found chromosomes (2B, 3B, 1R, 2R, 4R and 7R) containing regions where markers exhibited a distorted segregation pattern. In addition, we observed evidence for segregation distortion between pairs of loci caused either by a predominance of parental or recombinant genotypes.

**Conclusions:**

We have constructed a reliable, high-density DArT marker consensus genetic linkage map as a basis for genomic approaches in triticale research and breeding, for example for multiple-line cross QTL mapping experiments. The results of our study exemplify the tremendous impact of different DH production techniques on allele frequencies and segregation distortion covering whole chromosomes.

## Background

For scientists as well as commercial breeders the hexaploid man-made wheat-rye hybrid triticale (x *Triticosecale *Wittmack; 2n = 6 × = 42) is considered a promising crop with a broad genetic potential. Triticale is a partially outcrossing species and stands out due to a wide adaptation to abiotic stress conditions like salinated or acid soil, aluminium toxicity, drought, and waterlogged soils [1,2]. Furthermore, the cereal has attained importance as feed stock owing to a valuable composition of amino acids and a stable performance in less productive environments [3]. Providing raw material for the generation of bioenergy and biofuels, triticale produces more biomass for a comparable grain yield than other crops and, therefore, can increase the industrially useable biomass without increasing competition with food production on arable land [4].

Modern genomic approaches to enhance the breeding progress such as association mapping or genomic selection [5-7] require the availability of high-quality and high-density genetic linkage maps. For triticale, only one genetic map based on 73 DH lines and 356 markers with an average map density of 6.9 cM has been published so far [8]. Markers are, however, neither distributed homogenously among the different genomes (50.7% located on R genome) nor on the chromosomes. For example only two markers are located on each of the 1A, 4A, and 3B chromosomes. Therefore, a highly saturated genetic linkage map for triticale is urgently required to enable genomics research and knowledge-based breeding.

A prerequisite for the construction of genetic linkage maps is the availability of polymorphic molecular markers. Diversity Arrays Technology (DArT) markers [9] have been identified as a valuable tool in cereals and have been employed successfully to create linkage maps of the triticale parents wheat [10-14] and rye [15]. Badea et al. [16] have recently reported the development of a triticale-specific DArT array combining markers developed in wheat, rye and triticale.

Consensus maps are genetic maps that are created by a joint analysis of the data from several segregating populations and different approaches are available for the construction of such maps. In the joint data process, segregation data from individual populations are pooled and loci orders and genetic map distances are computed based on mean recombination frequencies and combined LOD scores (implemented in JoinMap^® ^4) [17]. Merged linkage maps not only provide a mean to assess associations among individual linkage maps but are also the basis for QTL studies in multiple segregating populations [18].

The phenomenon that alleles at a locus deviate from the Mendelian expectation has been defined as segregation distortion [19] and has been described in many species, for example maize [20], rice [21], tomato [22] and alfalfa [23]. Sample size and genotyping errors are some non-biological factors that can contribute to segregation distortion. Biologically, segregation distortion can be due to selection among gametes and/or zygotes [24]. If an allele at a locus diminishes gametic or zygotic fitness, then that locus and other loci linked to it will deviate from the expected Mendelian segregation ratio. Biological segregation distortion will, therefore, always effect a cluster of markers within the chromosomal region surrounding the segregation distortion locus [25-27]. In general, the contribution of different factors to segregation distortion may vary in different populations [21]. Several studies have identified QTL that affect DH production and these can lead to segregation distortion in regions surrounding these QTL [28]. For doubled haploid (DH) plant development several techniques are available which differ in (i) the sex of the source material (female vs. male gametes), (ii) the genesis of plantlets (zygotic embryos vs. embryoid bodies from microspores) and (iii) growth conditions in *in vitro *culture. The study of segregation distortion is, therefore, of particular interest in DH populations of diverse origin.

The objectives of this study were to (i) develop a high-quality saturated consensus linkage map combining DArT marker data from six triticale mapping populations, (ii) evaluate the colinearity and thus the reliability of the component maps by a comparison with the consensus map, and (iii) assess the presence and the extent of segregation distortion in the six triticale mapping populations developed by different approaches.

## Results

### Genetic diversity analysis

**Figure 1:**
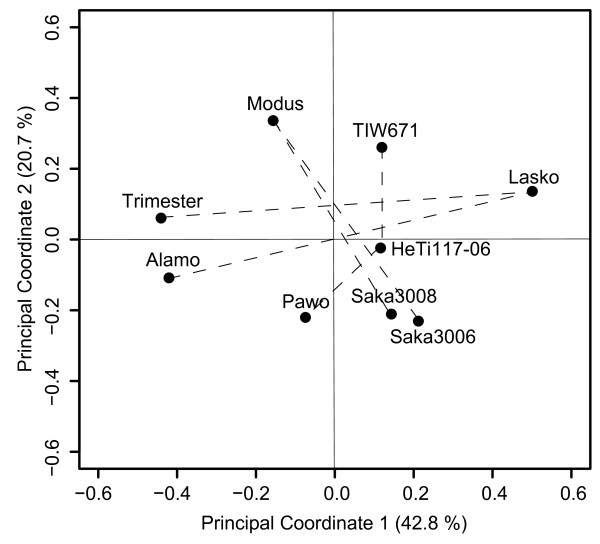
**Plot of the first two principal coordinates of the parents of the component populations**. Principal coordinate analysis of the nine parents of the populations, based on modified Rogers' distance estimates. Crosses between parental lines are indicated by dashed lines. The numbers in parentheses refer to the percentage of variance explained by the principal coordinate.

This study was based on six segregating populations derived from nine parental lines of which three were used as a common parent each contributing to two populations (Table 1). The DArT markers from the integrated map were used to assess the genetic similarity between the parental lines in a principal coordinate analysis based on the modified Rogers' distances of the individuals. The first two principal coordinates explained 42.8% and 20.7% of the total variation, respectively (Figure 1). The principal coordinate analysis revealed different degrees of relatedness but none of the parental lines was clearly separated from the others. The genetic distances among the six segregating populations revealed that populations F2_LxT and DH_LxA had the highest degree of genetic similarity (0.79) whereas populations EAW74 and DH07 were most distant (0.53) (Table 2).

**Table 1:** Description of the mapping populations

Pop^a ^code	Pop^a ^pedigree	Pop^a ^type	Pop^a ^size	Markers^b^
DH06	Modus × Saka3006	DH	131	1244
DH07	Modus × Saka3008	DH	120	1064
EAW74	HeTi117-06 × Pawo	DH	200	713
EAW78	HeTi117-06 × TIW671	DH	200	673
DH_LxA	Lasko × Alamo	DH	146	979
F2_LxT	Lasko × Trimester	F_2_	114	510

^a ^Pop = population.

^b ^Number of markers polymorphic in this population after quality pre-selection.

**Table 2:** Common markers and genetic similarity between mapping populations

	DH06	DH07	EAW74	EAW78	DH_LxA	F2_LxT
DH06		681	293	277	353	129
DH07	0.72		260	276	263	135
EAW74	0.57	0.53		254	244	104
EAW78	0.57	0.54	0.65		191	81
DH_LxA	0.65	0.64	0.66	0.64		204
F2_LxT	0.60	0.62	0.64	0.64	0.79	

Number of markers common between component maps is shown above the diagonal and genetic similarity between the component mapping populations below the diagonal.

### DArT marker linkage maps of individual populations

The component maps were constructed from six datasets containing between 114 (F2_LxT) and 200 (EAW74, EAW78) individuals and between 510 (F2_LxT) and 1244 (DH06) markers (Table 1). Out of these initial datasets 481 (F2_LxT) to 1209 (DH06) loci could be mapped on the 21 chromosomes (Table 3). The total lengths of the six resulting linkage maps varied between 1745.0 and 3270.9 cM. To estimate average marker densities, each group of co-segregating markers was considered as single marker (unique locus) to avoid artifacts leading to an overestimation of the actual values. Depending on the respective map length, single marker position counts from 435 (F2_LxT) to 755 (DH06) led to an average density of one unique marker locus every 4.0 (F2_LxT) to 5.2 cM (EAW78). With genetic map distances between unique loci varying from 0.1 to 111.6 cM, DArT markers were not evenly distributed along the chromosomes and in addition, showed cluster formation in different regions of the chromosomes. Ninety percent (2861 out of 3186) of the intervals between adjacent markers, however, were smaller than 10 cM and only 4.4% (140 out of 3186) exceeded 20 cM.

**Table 3:** Description of the component maps and the consensus map

	DH06				DH07				EAW74				EAW78				DH_LxA				F2_LxT				Consensus			
	Length^a^	Loci	Unique	Dens^b^	Length^a^	Loci	Unique	Dens^b^	Length^a^	Loci	Unique	Dens^b^	Length^a^	Loci	Unique	Dens^b^	Length^a^	Loci	Unique	Dens^b^	Length^a^	Loci	Unique	Dens^b^	Length^a^	Loci	Unique	Dens^b^
***Chromosome 1***																												
**A**	107.3	24	17	6.3	127.3	24	19	6.7	74.7	7	7	10.7	81.3	12	7	11.6	11.1	5	4	2.8	73.1	9	9	8.1	181.7	40	37	4.9
**B**	146.6	53	32	4.6	127.6	29	21	6.1	218.9	57	30	7.3	133.6	38	26	5.1	106.6	24	14	7.6	33.9	11	11	3.1	101.1	107	81	1.2
**R**	41.1	17	12	3.4	70.4	17	12	5.9	*	*	*	*	9.2	10	5	1.8	103.1	96	41	2.5	124.2	59	56	2.2	69.2	143	97	0.7
***Chromosome 2***																												
**A**	103.8	32	20	5.2	149.9	38	27	5.6	*	*	*	*	69.3	15	9	7.7	94.3	15	11	8.6	31.9	3	3	10.6	105.0	54	45	2.3
**B**	267.0	32	20	13.4	211.9	44	35	6.1	140.0	20	18	7.8	116.1	24	14	8.3	152.9	25	22	7.0	144.5	12	11	13.1	173.4	87	66	2.6
**R-1**	93.0	24	16	5.8	134.4	68	37	3.6	19.9	12	10	2.0	*	*	*	*	181.2	106	48	3.8	225.5	68	60	3.8	105.3	141	113	0.9
**R-2**	~	~	~	~	~	~	~	~	~	~	~	~	~	~	~	~	~	~	~	~	~	~	~	~	10.5	10	10	1.1
***Chromosome 3***																												
**A**	146.8	33	26	5.6	157.4	27	22	7.2	142.7	23	21	6.8	131.3	12	9	14.6	78.9	19	13	6.1	21.6	4	4	5.4	111.5	62	56	2.0
**B**	161.5	49	35	4.6	211.5	51	40	5.3	13.9	7	3	4.6	104.0	28	14	7.4	104.1	21	6	17.4	124.2	36	21	5.9	117.1	98	73	1.6
**R**	112.8	26	20	5.6	115.2	24	14	8.2	75.7	43	30	2.5	152.5	74	44	3.5	70.5	57	31	2.3	94.5	41	38	2.5	118.0	157	104	1.1
***Chromosome 4***																												
**A**	153.8	40	25	6.2	57.6	30	15	3.8	36.1	27	11	3.3	62.7	7	7	9.0	10.0	3	3	3.3	67.0	28	26	2.6	107.6	75	56	1.9
**B**	132.1	14	11	12.0	103.8	13	11	9.4	111.6	11	8	14.0	54.5	6	3	18.2	106.5	15	8	13.3	1.8	6	6	0.3	104.9	21	17	6.2
**R**	238.8	180	105	2.3	192.1	120	73	2.6	397.8	101	80	5.0	232.0	66	53	4.4	183.2	152	70	2.6	34.2	20	19	1.8	92.6	344	231	0.4
***Chromosome 5***																												
**A**	73.0	12	8	9.1	59.6	12	10	6.0	97.1	10	7	13.9	13.6	9	4	3.4	93.1	11	6	15.5	21.9	8	7	3.1	55.9	29	22	2.5
**B**	162.5	48	29	5.6	150.3	35	22	6.8	128.2	42	31	4.1	120.5	36	28	4.3	6.3	8	5	1.3	116.3	23	23	5.1	111.2	94	78	1.4
**R**	259.4	149	82	3.2	119.4	59	26	4.6	29.2	20	17	1.7	170.9	66	45	3.8	77.9	84	31	2.5	79.4	32	31	2.6	94.7	237	168	0.6
***Chromosome 6***																												
**A**	196.8	61	47	4.2	245.3	58	42	5.8	172.2	24	17	10.1	169.0	20	17	9.9	166.3	32	20	8.3	185.8	23	23	8.1	111.6	95	85	1.3
**B**	216.0	92	55	3.9	210.4	64	49	4.3	160.7	77	43	3.7	149.7	40	31	4.8	162.1	90	35	4.6	1.0	4	4	0.3	117.7	144	109	1.1
**R**	241.3	144	89	2.7	212.5	138	80	2.7	167.8	69	56	3.0	241.2	110	67	3.6	120.5	91	58	2.1	81.9	28	25	3.3	82.1	269	200	0.4
***Chromosome 7***																												
**A**	97.3	24	18	5.4	115.3	33	23	5.0	140.6	29	24	5.9	114.6	16	16	7.2	214.7	51	42	5.1	62.3	10	9	6.9	121.9	75	71	1.7
**B**	207.1	60	43	4.8	131.3	40	19	6.9	134.6	33	23	5.9	106.2	47	35	3.0	114.1	61	30	3.8	97.4	16	14	7.0	126.0	113	91	1.4
**R**	112.9	95	45	2.5	146.9	117	59	2.5	116.0	78	49	2.4	74.7	9	7	10.7	*	*	*	*	122.6	40	35	3.5	90.9	207	119	0.8

**ΣΣ**	**3270.9**	**1209**	**755**	**4.3**	**3050.1**	**1041**	**656**	**4.6**	**2377.7**	**690**	**485**	**4.9**	**2306.9**	**645**	**441**	**5.2**	**2157.4**	**966**	**498**	**4.3**	**1745.0**	**481**	**435**	**4.0**	**2309.9**	**2602**	**1929**	**1.2**

**Σ **A	704.7	146.7	109.2	6.5																					795.2	430	372	2.1
**Σ **B	900.6	240.3	156.5	5.8																					851.4	664	515	1.7
**Σ **R	879.3	451.7	279.3	3.1																					663.3	1508	1042	0.6

^a ^Chromosome length in cM.

^b ^Dens = Density; averaged distance between adjacent unique loci in cM.

* No linkage group obtained for this chromosome.

~ No second linkage group necessary for chromosome 2R in this population.

ΣΣ Sum of chromosome lengths, loci, unique loci, and averaged map density, respectively for each component map and the consensus map.

Σ Features of the component maps for the A, B, and R genome represent mean values over all component populations.

A comparison of the genetic maps with regard to the three triticale genomes, A, B and R, revealed that the A genome chromosomes showed the lowest marker coverage with an average number of 146.7 loci (109.2 unique) and an average marker distance of 6.5 cM. In contrast, the R genome chromosomes containing 451.7 loci (279.3 unique) and a map length of on average 879.3 cM featured the highest marker density with a mean genetic map distance of 3.1 cM between unique loci (Table 3).

A total of 81 (EAW78 and F2_LxT) to 681 markers (DH06 and DH07) were in common between pairs of component maps and we observed a high correlation (r = 0.72; P = 0.02) between the number of common markers and the genetic similarity between the populations (Table 2).

### A consensus genetic linkage map from the combined datasets

**Figure 2:**
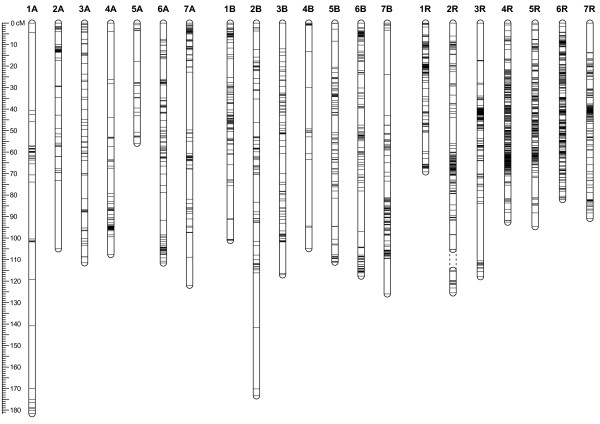
**Schematic illustration of the consensus map**. Unique loci are represented on their positions by horizontal lines across the chromosome. Chromosome 2R is divided into two linkage groups as indicated by dashed lines.

To construct the triticale DArT marker consensus linkage map, the datasets containing the markers mapped on the component maps were merged for a joint analysis. The final consensus map incorporated 2555 markers mapped to a total of 2602 loci of which 1929 loci (74.1%) were unique (Table 3 Figure 2). Among the 2602 mapped loci, 1553 were computed on the basis of information derived from at least two mapping populations and 53 loci were based on five or six mapping populations. A subset of 47 out of the 2555 DArT markers (1.8%) mapped to two different loci in the consensus map. In more than 80% of the cases the genetic map positions of the different copies of these markers were found to be located either on a different chromosome within the same genome, or on a homeologous chromosome. The consensus map spanned a total length of 2309.9 cM resulting in an average marker density of one unique locus every 1.2 cM. Chromosome sizes ranged from 55.9 cM (chromosome 5A) to 181.7 cM (1A). Chromosome 2R was composed of two linkage groups 2R-1 and 2R-2 which could not be integrated applying the chosen mapping parameters. Linkage groups 2R-1 and 2R-2 covered 105.3 cM and 10.5 cM, respectively, with a total of 151 mapped loci of which 123 were unique. On the consensus map the number of loci per chromosome ranged from 21 (17 unique) on 4B to 344 (231 unique) on 4R with an average of 123.9 loci (91.9 unique) per chromosome. The average marker density based on unique loci ranged from 0.4 (4R, 6R) to 4.9 cM (1A). In the consensus map the largest gap between single marker positions spanned 36.2 cM and 99% of the intervals were smaller than 10 cM. Also in the consensus map, clustering of DArT markers became evident in different regions of the chromosomes and to a greater extent on chromosomes of the A and B genomes (Figure 2). Furthermore, markers were not distributed equally among the A, B and R genomes. The number of mapped loci increased from the A genome with 430 (372 unique) to 664 (515 unique) on the B genome. The R genome featured the highest number of mapped loci totaling 1508 loci (1042 unique) with a total length of 663.3 cM and also the highest genetic map density among the triticale genomes, with an average marker density of 0.6 cM. We found that 16 DArT markers originating from the wheat D genome could be mapped on various chromosomes of the consensus map. Key data of the consensus map useful for further research including marker names, mapped chromosomes and positions are available as additional file (Additional file 1).

### Comparison of the consensus map with the individual linkage maps

**Figure 3:**
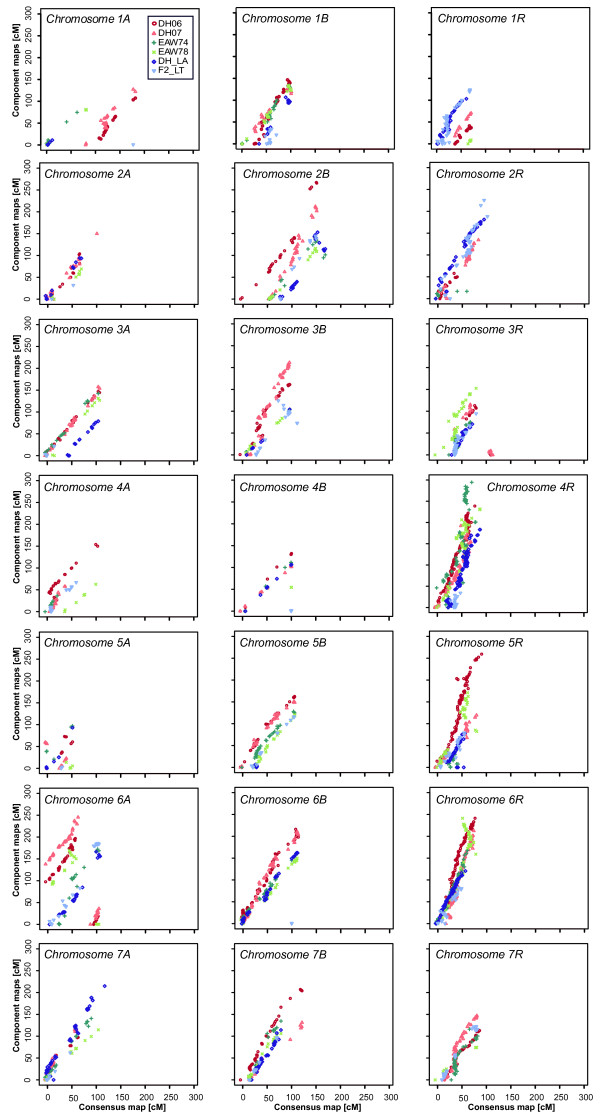
**Comparison of loci positions in component maps and the consensus map**. DH06, DH07, EAW74, EAW78; DH_LxA, and F2___LxT are indicated by red circles, pink triangles, green crosses, light-green exes, blue diamonds and light-blue triangles, respectively.

To evaluate the quality of the consensus linkage map, we assessed the consistency of locus order between the consensus map and the component maps. For this comparison, the marker positions of the consensus map were plotted against the positions in the individual component maps separately for each chromosome (Figure 3). In general, locus order was well maintained between the consensus linkage map and the component maps. We observed few groups of loci, however, for which the colinearity between the component maps and the consensus map was uneven. On chromosome 1A of the DH07 map, for example, loci in the interval between 35-50 cM showed the same sequence but reverse orientation compared to the consensus map. Similarly, this was observed for chromosome 3B of the F2_LxT population (interval 90-125 cM) and for chromosomes 6A and 6R of population EAW78 (intervals 150-170 and 200-250 cM, respectively). For chromosomes 3R and 6A of the DH06 and DH07 specific maps the colinearity plots revealed that loci on the 0-10 cM and 0-40 cM intervals, respectively, were located at the opposite end of the chromosome compared to the consensus map. The locus sequence in these intervals, however, was well conserved. Highly dense marker regions of the consensus map, mainly found on chromosomes of the R genome, exhibited partial marginal shifts in locus order compared to the component maps. On a more global level, the colinearity plots revealed disparities in length between the component maps and the consensus map. In the consensus map identical pairs of loci resulted in shorter genetic map distances condensing most chromosomes.

### Segregation distortion

**Figure 4:**
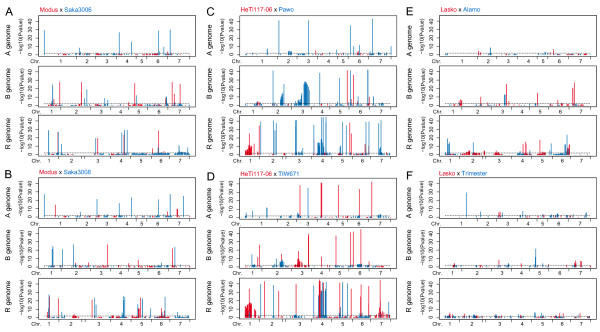
**Segregation distortion of loci in the component maps based on the consensus map**. Segregation distortion in favour of one parental allele is indicated in red or blue, respectively. Populations (A) DH06, (B) DH07, (C) EAW74, (D) EAW78, (E) DH_LxA, (F) F2_LxT. The dashed horizontal line indicates the significance threshold (P > 0.01).

**Figure 5:**
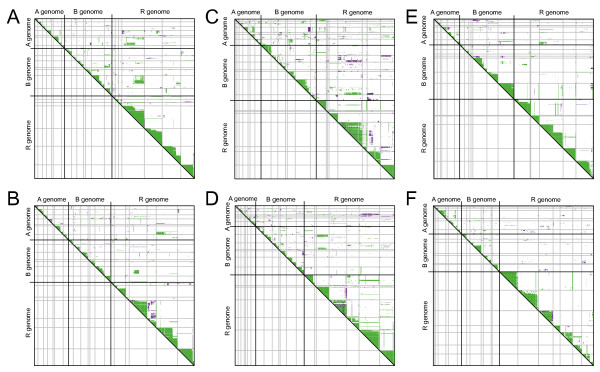
**Segregation distortion caused by epistatic interactions**. Significant segregation distortion (P < 0.01) for pairs of loci is shown in green (parental genotypes dominating) and in purple (recombinant genotypes dominating). Lines separate genomes (black) and chromosomes (grey). Both axes contain the markers segregating in the respective population, with the markers in the order of the chromosomes from 1 to 7. Populations (A) DH06, (B) DH07, (C) EAW74, (D) EAW78, (E) DH_LxA, (F) F2_LxT.

Among the 2602 loci mapped on the consensus map, 886 (34.0%) showed distorted segregation (P < 0.01) in at least one of the individual mapping populations, whereas only 311 of these loci (11.9%) deviated in two or more populations. The rate of distorted markers in the individual populations averaged 19.9% and totalled 11.5% (156 out of 1352) in DH06, 8.3% (98 out of 1187) in DH07, 33.6% (329 out of 980) in EAW74, 34,3% (357 out of 1042) in EAW78, 13.2% (145 out of 1102) in DH_LxA and 27.2% (161 out of 592) in F2_LxT. On the level of single chromosomes, the proportion of markers deviating from the expected segregation ratio ranged from 0 to 100%. Markers on chromosomes 5A (DH06) and 1A, 3A, 7A, 4B and 7R (DH_LxA) followed the expected 1:1 segregation ratios, whereas all markers on chromosome 1R (EAW74) showed a significant deviation. As shown in Figure 4, there were several chromosomes with large regions in which the markers exhibited a distorted segregation pattern, namely in population DH06 on chromosome 7R, in populations EAW74 and EAW78 on chromosomes 2B, 3B, 1R, 4R and 7R, and in population DH_LxA on chromosome 2R. The distorted regions exhibited a pattern with slight segregation distortion at the flanks, which increased towards more highly distorted loci in the centre. In addition, we observed evidence for segregation distortion between pairs of loci caused either by a predominance of parental or recombinant genotypes (Figure 5).

## Discussion

Genome-wide high-density genetic linkage maps specific to a species are both useful and essential for a number of reasons. These include the identification of marker-trait associations via linkage analysis and association mapping, for isolation of genes by map-based cloning, for comparative mapping and for exploration of genome organisation [29,30]. In our study, we have created the first high-density consensus genetic linkage map integrating DArT marker data from six triticale mapping populations.

### Evaluation of method and test conditions

In this study, the integrated genetic linkage map was calculated by using the joint data approach and regression mapping algorithm of JoinMap^® ^4 [17]. For effective linkage map pooling (and bridging) the individual mapping populations should share some common genetic bases (populations possessing a common parent for example) or common statistics (similar linkage information), but genetic map construction also relies on genome variation at loci detectable by molecular markers [31]. Our study was based on segregation data from six partially connected mapping populations derived from nine triticale elite breeding lines (Table 1). In reference to the first two principal coordinates the parental lines of each cross were derived from distinct quadrants (Figure 1), generating a maximum amount of genetic diversity. On the other hand, genetic similarity values between individual populations and the amount of markers found to be in common between them indicate a certain connectedness between the populations (Table 2). These findings clearly show that the plant material underlying this study meets both requirements for linkage map construction and pooling. That is, sufficient variation for polymorphic segregation data as well as common genetic bases for a statistically firm integration.

Genetic linkage maps are established by heuristical algorithms and statistical approaches and thus, have an inherent statistical error. This holds true for the estimation of the recombination frequencies as well as for the integration of data from several populations. Sample size is crucial for genetic map construction as it affects the power of linkage detection and the accuracy of recombination fraction estimation [31]. Random variation and potential biological variation can cause differences in estimated pairwise distances between individual populations, particularly if populations are of small size [17]. We investigated six mapping populations incorporating 114 to 200 progenies, and a total sample size of 911 individuals (Table 1) which is similar to recent studies in rye [32], sorghum [33], red clover [34] and barley [35]. We observed differences in the estimation of recombination frequency between identical pairs of markers in different populations [31] and found that for intervals up to 10 cM this heterogeneity increased successively and declined again for larger genetic distances (Additional file 2). The extent of heterogeneity between our individual mapping populations was expected due to both the effect of random sampling and biological variation which is occurring regardless of common genetic bases among populations.

In conclusion, the mapping populations used in our study were well suited for reliable consensus linkage map building. However, our results also highlight the fact that consensus maps always constitute a compromise which must be kept in mind.

### Distribution of DArT markers

During the development and assessment of DArT markers in triticale, a larger number of polymorphic markers originated from the rye genome and more wheat markers from the B genome [16]. With 57.8% of markers mapped to the R genome, 25.5% to the B genome and only 16.5% to the A genome (Table 3) we could verify these results in an applied mapping experiment with a large number of populations and individuals. Similar results have been found in studies of wheat for DArT [10-12] as well as other marker types [36-39]. We, therefore, conclude that the bias observed in our study is not attributable to the mapping individuals or the type of marker. Instead it may be due to the design of the triticale DArT array and the number of markers originating from the different genomes, but is also likely to reflect the different polymorphic nature of the A, B, and R genomes [16]. In our study, 1.8% of the DArT markers mapped to two different loci on the consensus map, but never on the same chromosome (Additional file 1). In terms of the ratio of markers that occur in a multicopy manner, our results agree well with those reported for hexaploid wheat (2%), barley (1.4%) and sorghum (1.8%) [10,33,35]. This may be attributable to the polyploid nature of hexaploid triticale having an impact on the accuracy of DArT markers due to alternative binding sites on homeologous chromosomes or may be ascribed to paralogous sequences. Molecular markers are known for their tendency to cluster, caused either by an unbalanced distribution of recombination events along chromosomes or an unequal representation of chromosomal regions on the genotyping array [33]. In accordance with this expectation we found that DArT markers clustered in several chromosomal regions (Figure 2). A possible explanation for regions with higher marker density on chromosomes could be that recombination occurs more frequently in gene-rich regions [40] which are present in clusters comprising physically small chromosomal regions and account for only 5-10% of the wheat genome [41,42]. The observed gaps in the consensus map may, on the other hand, be caused by identity-by-descent of the parental genotypes in these genomic regions. Taken together, clustering of tightly linked loci and gaps with low marker density in the consensus map either reflect the genetic situation in triticale or are due to specific properties of the applied DArT markers (e.g. complexity reduction step, or redundant clones). Further research including alternative high-density marker systems, e.g. SNPs, will help to addess this question.

### Consensus map features

Marker coverage and genetic map density are influenced by many criteria such as genome length, number of markers, distribution of markers and crossovers in the genome, mapping population size and mapping strategy [31]. As a result of the integration of datasets from six mapping populations our final triticale consensus map incorporated 2602 loci (74.1% unique) covering 2309.9 cM (Table 3). The previously published triticale genetic linkage map [8] comprised 356 markers (AFLP, RAMP, RAPD, SSR) and spanned 2465.4 cM. Thus, despite different marker types being used, these results agree well in regard to computed map lengths for the triticale genome.

Likewise our results are in good accordance with the reported map length of 2383 cM based on 339 DArT markers for the related hexaploid bread wheat (AABBDD) [10]. In our study the linkage groups of the A and B genome together covered 1646.6 cM and those of the R genome 663.3 cM. Contrary to these results, the published genetic maps of durum wheat (AABB) [12] and rye (RR) [15] based on DArT markers spanned 2022 and 3144.6 cM, respectively. As mapping functions were similar in the studies, the disparities may be explained by a different number of markers mapped and/or the different mapping algorithms applied. Referring to unique markers, many appeared redundant in component maps and were just 0.1 cM apart after integration. This raises the question whether these loci are really distinct from each other or are just a product of statistics and therefore should not be regarded as unique in further QTL mapping studies.

The average density in our consensus map was one unique marker every 1.2 cM, and 98% of all intervals between adjacent loci were smaller than 10 cM. This is sufficient for QTL mapping and many modern genomics approaches [43]. It may, however, be worthwhile amending larger gaps by target-oriented employment of additional markers. Thus, we conclude that through integration of datasets from six mapping populations we were able to improve both the density and the quality of the component maps up to the final high-density DArT marker triticale consensus map.

### Colinearity

One possible method to assess the quality of a consensus map is to compare the locus arrangement of the consensus map, which was optimised at the multi-population level, with the arrangement of loci in the component maps (each one optimised independently) [35]. A consistent order is hypothesised if the markers identify identical chromosomal locations and if there are no incorrect or missing scores [44]. In addition, this colinearity comparison can identify chromosome rearrangements in individual populations. Our tests for colinearity resulted in an overall good consistency affirming the high quality of our consensus map (Figure 3). We also found, however, regions where groups of neighbouring loci showed identical order of loci but inversion within a linkage group or even positioning of a region with conserved order at the opposite end of the chromosome. Such inconsistencies were reported for other species including red clover [34] and sorghum [33]. In triticale, chromosomal rearrangements are known to occur [45]. Thus, these inversions of marker order or positioning could reflect real genetic events such as small chromosome rearrangements or, as they occur mostly after gaps, they could also be caused by statistical uncertainty due to many weak linkages contributing to the adjustment. Furthermore, marginal shifts in locus order were found in regions with highly dense markers. Similar results were reported before in several mapping experiments [32-35,46]. Despite a certain heterogeneity of recombination frequencies between mapping populations this must mainly be attributed to the dependency of estimated gene orders on sample size [31]. Especially for the high-density regions, extremely large mapping populations would be required to resolve the correct order of markers.

The colinearity plots revealed that respective linkage groups were generally longer in the component maps than in the consensus map and this effect was even larger in denser linkage groups. The application of different algorithms (maximum likelihood or regression approach) during component and consensus map construction has been reported to affect map lengths, despite the same mapping function [32,34,46]. Another explanation could be that the condensed map length may be the intended outcome of the addition of more markers during the integration process [32].

### Segregation Distortion

Segregation distortion is known to strongly impact genetic map construction and QTL mapping [26,27] but distorted markers may also be beneficial for QTL mapping if handled properly [47]. Whereas highly deviating markers cannot be placed in the respective component maps, they can be included in the consensus map through integration of unbiased information available from other populations without segregation deviation. Our experimental design with multiple segregating populations thus offered an excellent basis for the evaluation of segregation distortion and the mapping of segregation distortion QTL.

The component maps of two populations were completely lacking certain linkage groups (2A and 1R in EAW74, and 2R in EAW78) (Table 3). As the three linkage groups were well covered in other populations and the same markers were also positively scored in the populations with the lacking chromosomes we can exclude a scarcity of markers. The majority of the markers on these chromosomes (79 and 100% of the markers mapped on chromosomes 2A and 1R in the consensus map, respectively), however, showed significant segregation distortion in population EAW74. This illustrates the possible consequences of segregation distortion which not only affects genetic map distances and ordering of loci, but can even result in complete chromosomes being absent from genetic maps.

The populations underlying our study were five DH and one F_2 _population. The DH populations were produced by different methods, either by maize pollination of the oocytes (DH06, DH07, DH_LxA) or by microspore culture (EAW74, EAW78) and were not pre-selected for any trait. We observed segregation distorted regions caused by biological factors (regions of distorted markers which had the same skew direction, distinguishable from deviating loci scattered apart along the chromosomes which are likely due to genotyping errors or non-functional markers) only in DH populations and mainly in microspore culture derived populations (Figure 4). The patterns observed in segregation distortion regions can be explained by both the distance between the markers and the segregation distortion loci linked to them and the effects of those loci [48]. Our test for segregation distortion in the microspore derived populations resulted in clusters of distorted markers on chromosomes 2B, 3B, 1R, 4R, and 7R. In studies of wheat DH populations, chromosome 2B was reported to harbour QTL responsible for green spot initiation and plant regeneration [49] and a different type of *in vitro *culture response in anther culture [28]. Furthermore, QTLs located on chromosomes 1R, 4R and 7R were previously reported to have an effect on the yield of green plantlets from anthers in culture and embryo induction (7R) in triticale [8]. Segregation distortion on chromosome 7R was, besides EAW74 and EAW78, also observed in DH06. This chromosome has been implicated in the selection of zygotes or female gametes in rye [50]. To our knowledge no QTL affecting segregation distortion have been described for chromosomes 3B (EAW74 and EAW78) and 2R (DH_LxA) yet. Due to the consistent occurrence in both microspore derived DH populations we assume that this region located on chromosome 3B harbours a novel QTL responsible for *in vitro *or androgenetic response in triticale.

Epistasis refers to interactions between two or several loci [51,52] and has recently been shown to contribute to segregation distortion [53,54]. In accordance with this we observed epistatic interactions involved in segregation distortion (Figure 5). These epistatic interactions point towards selection for specific allele combinations for *in vitro *or androgenetic response in triticale.

The DH technology has become an indispensable part of both research and breeding of triticale and many other agronomically important crops. Depending on the effect of the segregation distortion locus, it can influence the allele frequencies on an entire chromosome, including all genes involved in the expression of important agronomic traits. If the segregation distortion locus and the QTL for the agronomic trait are linked in repulsion, the agronomic QTL will be underrepresented or in the most extreme case absent from the population. The same holds true for the introgression of traits, if the QTL are by chance located on chromosomes harbouring segregation distortion loci. The relatively high number of segregation distortion loci identified in our study highlights this problem both for research and applied breeding. Further characterisation or even identification of the nature of segregation distortion loci may facilitate solving these issues.

## Conclusions

We have constructed the first DArT marker consensus genetic linkage map for triticale by integrating segregation data from six mapping populations. The colinearity of the consensus map was well maintained and it is, therefore, sufficiently reliable for use in multiple-line cross QTL mapping experiments and in addition may serve as a reference for genetic maps created from other triticale germplasm. The results of our study underpin the impact of different DH production techniques on segregation distortion and allele frequencies covering whole chromosomes. In this context we identified a previously unknown region located on chromosome 3B likely to be responsible for *in vitro *or androgenetic response in triticale. Our results imply that caution must be exerted when DH populations are utilised in research or applied breeding.

## Methods

### Plant material and DNA extraction

This study was based on 911 triticale lines (*Triticosecale *Wittmack L.) from six mapping populations derived from nine parental lines (Table 1). Populations DH06 (131 individuals), DH07 (120), EAW74 (200), EAW78 (200), and DH_LxA (146) were doubled haploid (DH) lines whereas the F2_LxT population (114 individuals) was an F_2 _population. Leaf tissue was harvested around five leaf stage from single plantlets and dried in silica gel. High-quality genomic DNA for genotyping was isolated from 20-25 mg of dried leaf tissue according to a modified CTAB method [55] and adjusted to a concentration of ca. 50 ng/μl for marker analysis.

### Marker and molecular analysis

Marker data for genetic mapping were obtained by DArT genotyping of all samples [9]. DArT genotyping of the individuals used in this study was carried out by Triticarte Pty Ltd, Yarralumla, ACT, Australia [56] with the current triticale array. The nine parental varieties were also DArT genotyped as described above. Associations among the nine parental lines were analysed by applying a principal coordinate analysis [57] based on the modified Rogers' distances of the individuals [58]. Similarity among the populations was estimated as one minus the modified Rogers' distances among the populations. Segregation distortion was calculated based on the *P *values obtained by a chi-square test and the parent contributing the allele with the higher frequency is indicated in Figure 4. To identify loci with epistatic interactions causing segregation distortion, we tested all possible pairs of markers using Fisher's exact test. Principal coordinate analysis and segregation distortion computations were performed with the software packages Plabsoft [59] and R [60], respectively.

### Genetic linkage map construction

For the construction of the genetic linkage maps, DArT markers polymorphic in a population were transformed into genotype codes according to the score of the parents. For quality filtering a pre-selection with regard to their segregation ratio was performed. Markers which significantly (P ≤ 0.001) deviated from the expected 1:1 (DHs) and 3:1 (F_2_) ratio in a chi-square test were excluded from further analyses. The genetic linkage maps were constructed with the software JoinMap^® ^4.0 [17]. Markers were assigned to linkage groups applying the independence LOD (logarithm of the odds) parameter with LOD threshold values ranging from 2.0 to 20.0. The test for independence is not affected by segregation distortion which allowed for the liberal level of significance in terms of deviation. Certain ungrouped markers were added to groups on a by-case basis according to the indicated strongest cross link (SCL) LOD values. Chromosome names and orientation were assigned to linkage groups based on a subset of markers in each linkage group for which the positions have recently been published ([10,15]; chromosome assignment by Triticarte Pty Ltd, Yarralumla, ACT, Australia [56]). The available information on the position of some of the DArT markers also allowed us to link chromosome regions that appeared unlinked at the LOD = 2.0 level. Markers causing suspect linkages due to an estimated recombination frequency > 0.6 were excluded in the particular population. During the calculation of the individual maps from the six populations the locus order within chromosomes and estimation of recombination frequencies were established employing the provided maximum likelihood algorithm with modified calculation settings. For an adjusted map order optimisation, chain length and stopping criterion were extended to 5000, the cooling control parameter was decreased to 0.0001. The maximum likelihood algorithm was used to establish the map order of the markers within a defined linkage group and the genetic distances in centimorgan (cM) values were output converted with Kosambi's mapping function [61]. After each run post-mapping quality filtering tools provided by JoinMap^® ^4 for the maximum likelihood method such as the plausible position matrix and the fit and stress monitoring were studied and markers causing a poorer fit were excluded. For the construction of the consensus linkage maps quality filtered data sets from individual populations related to the same chromosomes were joined together in one data set. In JoinMap^® ^the calculations of consensus maps are based on mean recombination frequencies and combined LOD scores of pairwise data from multiple populations. To screen for deviant pairs the heterogeneity test using a standard G^2^ statistic was used. In order to be able to exclude differences more likely to be due to the effect of random sampling or technical or statistical failure, and hence provide a basis for adequate linkage map pooling without significant differences as postulated by [31], pairs of loci highly deviating in their estimated recombination frequencies (P ≤ 0.001) were excluded from computation of the consensus linkage map. Thus, loci were not forfeit but placed more trustable by the use of multi-locus models.

Locus order and map distances were calculated using the regression mapping approach with the following settings: recombination frequency used < 0.49, LOD > 1.0, goodness-of-fit jump threshold = 5.0, number of added loci after which to perform a ripple = 1, mapping function = Kosambi, and third round = yes. After each run the post-mapping quality filtering tool indicating the mean chisquare-contributions was studied and markers causing a poorer fit were excluded. The graphical representation of the map was drawn using MapChart software [62].

## Authors' contributions

KVA carried out the mapping analyses, performed parts of the statistical analyses and drafted the manuscript. JCR participated in the design of the study and edited the manuscript. HPM and VH developed the DH06 and DH07 populations. EAW developed the EAW74 and EAW78 populations. TM developed the DH_LxA and F2_LxT populations. TW participated in the design of the study, performed parts of the statistical analyses and helped to draft the manuscript. All authors read and approved the final manuscript.

## Supplementary Material

Additional file 1 **Locus positions on the consensus map**. Excel spreadsheet containing a list of all consensus map loci. Data include (i) the locus referred to as marker name plus chromosome it was derived from (NA not available), (ii) chromosome and position the locus was mapped to and (iii) multicopy marker details. Click here for file

Additional file 2 **Heterogeneity of distances between unique loci among component populations**. Heterogeneity was tested by comparing observed numbers of recombinants (calculated from recombination frequency and LOD score) in the individual component populations with the expected numbers based on the mean recombination frequency [17]. P values denote values of the G^2^ statistic for each pair of loci summed up over all component populations contributing information for this pair. Respective map distances are based on the consensus map. Click here for file
